# Supplementary material for: From sequence to enzyme mechanism using multi-label machine learning
Source: BMC Bioinformatics. 2014 May 19;15:150. doi: 10.1186/1471-2105-15-150 (PMC4229970; doi:10.1186/1471-2105-15-150)
Supplement: Additional file 2 — Java code of ml2db. Additional file ml2db_code.tar.gz contains the Java source code to run the multi-label machine learning experiments and save the results to database. The code’s Javadoc is included. [file 1471-2105-15-150-S2.zip › additional file 2/ml2db/utils/doc/uk/ac/standrews/utils/main/package-tree.html]

uk.ac.standrews.utils.main Class Hierarchy


JavaScript is disabled on your browser.


- Overview
- Package
- Class
- Use
- Tree
- Deprecated
- Index
- Help

- Prev
- Next

- Frames
- No Frames

- All Classes

# Hierarchy For Package uk.ac.standrews.utils.main

Package Hierarchies:

- All Packages

## Class Hierarchy

- java.lang.Object
  - uk.ac.standrews.utils.main.EntrezUtils
  - uk.ac.standrews.utils.main.FileUtils
  - uk.ac.standrews.utils.main.Initialised (implements uk.ac.standrews.utils.main.Initialisable)
  - uk.ac.standrews.utils.main.LoggerCreator
  - uk.ac.standrews.utils.main.LogUtils
  - uk.ac.standrews.utils.main.NumberUtils
  - uk.ac.standrews.utils.main.PathUtils
  - uk.ac.standrews.utils.main.stringutils.Pool (implements java.io.Serializable)
    - uk.ac.standrews.utils.main.BooleanArrayPool
  - uk.ac.standrews.utils.main.PropertiesUtils
  - uk.ac.standrews.utils.main.ReflectionUtils
  - uk.ac.standrews.utils.main.RegExpUtils
  - uk.ac.standrews.utils.main.Scripts
  - uk.ac.standrews.utils.main.SystemUtils
  - uk.ac.standrews.utils.main.TimeUtils
  - uk.ac.standrews.utils.main.Utils

## Interface Hierarchy

- uk.ac.standrews.utils.main.Initialisable

- Overview
- Package
- Class
- Use
- Tree
- Deprecated
- Index
- Help

- Prev
- Next

- Frames
- No Frames

- All Classes
